# Immune Network Modeling Predicts Specific Nasopharyngeal and Peripheral Immune Dysregulation in Otitis-Prone Children

**DOI:** 10.3389/fimmu.2020.01168

**Published:** 2020-06-11

**Authors:** Matthew C. Morris, Timothy J. Chapman, Michael E. Pichichero, Gordon Broderick

**Affiliations:** ^1^Center for Clinical Systems Biology, Research Institute, Rochester General Hospital, Rochester, NY, United States; ^2^Center for Infectious Diseases and Immunology, Research Institute, Rochester General Hospital, Rochester, NY, United States; ^3^Department of Biomedical Engineering, Rochester Institute of Technology, Rochester, NY, United States

**Keywords:** pediatric population, systems biology, otitis, immune signaling, numerical models, regulatory logic, immune homeostasis

## Abstract

Acute otitis media (AOM) pathogenesis involves nasopharyngeal colonization by potential otopathogens and a viral co-infection. Stringently-defined otitis prone (sOP) children show characteristic patterns of immune dysfunction. We hypothesized that otitis proneness is largely a result of altered signaling between immune components that are otherwise competent, resulting in increased susceptibility to infection by bacterial otopathogens. To test this, we constructed a regulatory immune network model linking immune cells and signaling elements known to be involved in AOM and/or dysregulated in sOP children. The alignment of immune response mechanisms with data from *in vivo* and *in vitro* experimental observations produced 82 putative immune network models, each describing variants of immune regulatory networks consistent with available observations. Analysis of these models suggested that new measurements of serum levels of IL-4 and CXCL8 could refine competing models and resulted in the elimination of 38 of the models. Further analysis of the remaining 44 models suggested specific deviations in the predicted regulation of nasopharyngeal and peripheral immunity during response to AOM. Specifically, immune responses active in sOP children during AOM were characterized by early and constitutive activation of pro-inflammatory signaling in the nasopharynx and a Th2- and Treg-dominated profile in the periphery. We conclude that sOP children have altered regulation of key immune mediators during both health and pathogenesis. This altered regulation may be amenable to therapeutic intervention.

## Introduction

Otitis media (OM), frequently referred to as an ear infection, is among the most common childhood illnesses, with some 5 million cases annually in the US (1). OM is a leading cause of acquired deafness (2, 3), and 21,000 children are estimated to die from complications of OM annually worldwide (4). The nasopharyngeal (NP) mucosal compartment is the site of bacterial otopathogen colonization. Therefore, the local NP innate and adaptive immune response is critical for limiting invasive bacterial dissemination to other anatomical sites. Progression from asymptomatic NP carriage to acute disease is almost always associated with a viral upper respiratory infection (URI) (5). Systemically, circulating concentrations of the immune mediators S100A12 and IL-10 change in the context of ICAM-1 during an infection, and these changes are specific to the causative otopathogen (6). From analyses of nasal lavage samples during both health and illness, defective inflammatory cytokine production has been recognized as a characteristic of otitis-prone children (7). In the particular case of infection by *Streptococcus pneumoniae* (Spn), neutrophil infiltration and inflammatory cytokine production in the NP increase proportionally to bacterial burden (8). Heightened inflammatory responses during a precipitating viral URI have been shown to influence the likelihood of progression to AOM, with both cytokine production (9) and tissue injury (10) showing significant predictive associations.

Since 2006, our group has conducted a prospective, longitudinal study of AOM seeking to understand immune mechanisms responsible for infection proneness. In our studies, children were defined as stringently otitis prone (sOP) if they experienced 3 separate AOM infections within 6 months or 4 within a 12-month time frame. Children experiencing fewer AOM infections were defined as non-otitis prone (NOP) (1). All AOM events were confirmed by tympanocentesis and microbiological identification of the instigating bacterial otopathogen [most commonly *Streptococcus pneumoniae, Haemophilus influenzae*, or *Moraxella catarrhalis* (1)]. Other groups studying OP children have identified candidate genes in various immunological pathways (11), but those studies did not restrict the definition of OP to cases where microbiologic confirmation occurred. It is probable that a requirement for microbiologic proof of AOM refines the study population to allow clearer outcome differences during immunologic studies. Indeed, with this strict definition, we have identified immune dysfunctions in both nasal lavage and peripheral blood. During an AOM episode, sOP children produce reduced quantities of innate pro-inflammatory cytokines and epithelial cell repair enzymes in nasal lavage (12). In the peripheral blood, sOP children display poor otopathogen-specific antibody responses (13–15) and memory B cell generation (16), and defects in memory CD4 T cells (17). In particular, the importance of Th17 function in protection against pneumococcal infection is well-documented in both mice and humans (18–21). In our studies, Th17 cells from sOP children were observed to have deficient responses to pneumococcal components *in vitro*, but these deficiencies could be rescued by exogenous Th17-promoting cytokines (22). This suggests a failure of regulatory immune signaling without underlying cellular defects. We have also found that repercussions from immune dysregulation in sOP children were not limited to causing increased susceptibility to AOM: these children more often failed to generate protective antibody responses to routine pediatric vaccines (23, 24), broadening the significance of their immune deficits.

The interplay between these factors—the normal course of response to an infection, environmental, and genetic factors, as well as infection history—present challenges to the development of a mechanistic understanding of recurrent AOM. Unlike traditional statistical methods, systems biology techniques allow the integration of observations from different sources and experimental contexts, and are robust to missing data. We hypothesized that otitis proneness is largely a result of altered signaling between immune components that are otherwise competent, resulting in a state of increased susceptibility to NP colonization and infection by otopathogens. To test this, a step-wise discovery approach was used: (1) a review of published literature concerning pediatric immune responses to bacterial AOM for identification of immune system components involved in AOM pathogenesis and resolution; (2) construction of regulatory network models linking immune cell signaling elements known to be involved in response to otitis media and/or dysregulated in sOP children; (3) alignment of regulatory parameters for the network models with available data from *in vivo* AOM episodes and *in vitro* peripheral blood mononuclear cell (PBMC) experiments during health, at onset of AOM and after recovery in sOP and NOP children; and (4) analysis of the predicted dynamic behavior of the immune system during AOM pathogenesis and recovery to discover points of divergence between the immune response to AOM infection in sOP and NOP children. In addition to recapitulating Th1 and B cell dysfunction we had previously described (22, 25), these simulations additionally predicted a general Th2- and Treg-dominated immune profile in the systemic compartment in sOP children. In the nasopharynx, model predictions of response to bacterial infection suggested early and constitutive activation of pro-inflammatory signaling in sOP children. Modeling also highlighted TGFβ and viral infection history as potential contributors to the establishment and maintenance of the sOP phenotype.

## Materials and Methods

### Source of Clinical Samples

Our longitudinal, prospective study design to collect clinical samples has been previously described (26, 27). Children were from mostly middle-class suburban homes in Rochester, NY. Enrolled subjects attended healthy visits at 6, 9, 12, 15, 18, 24, and 36 months of age, as well as illness visits. Blood and nasal lavage samples were routinely provided at these visits as part of the study protocol. sOP children were defined as those who experienced at least 3 distinct episodes of AOM within a 6-month time span or at least 4 episodes of AOM within a 1-year time span. All AOM episodes were clinically diagnosed and confirmed by tympanocentesis sampling of middle ear fluid to identify causative otopathogens. Tympanocentesis eliminated the potential for contamination of the infection prone cohort by children who were inaccurately diagnosed, as is frequently the case (28, 29). NOP children were defined as those with absent or one to two AOM episodes in time frame of 6–36 months of age.

### Immune Network Model Assembly

**Figure 1:**
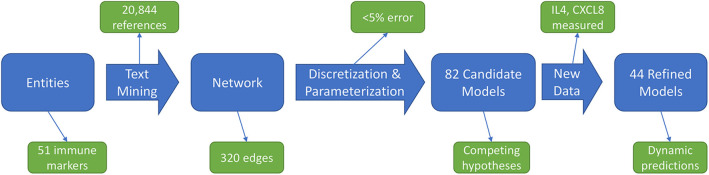
Workflow overview. An initial immune cell and molecular entities list is used to mine published scientific literature for mechanistic statements, yielding an immune network architecture. Discretized, qualitative descriptions of experimental data are then used to discover parameters for candidate models. Analysis of these candidate models points to informative experiments to refine the model pool through generation of new data.

The immune signaling network model was assembled after reviewing published literature concerning AOM and otitis proneness to identify important components of the immune system, namely cell populations and signaling molecules, involved in susceptibility to recurrent otitis media. The Pathway Studio database (Copyright © 2020 Elsevier Limited except certain content provided by third parties. Pathway Studio is a trademark of Elsevier Limited, Amsterdam) was then queried to identify mechanistic interactions linking these immune components using the natural language processing (NLP) engine MedScan (30) to mine the full text of 3.5 million peer-reviewed publications and an additional 24 million PubMed abstracts. The authors verified MedScan interpretation of the reported immune regulatory mechanisms. The full workflow of network generation, parameterization, and model refinement is summarized in Figure 1.

### Immune Model Parameterization

Immune network response was represented using a discrete logical formalism (e.g., if expression of protein A is High, then expression of protein B should become Low) designed to qualitatively recapitulate the dynamic behaviors of immune regulation typically described using more complex continuous kinetic models [(31–35); i.e., expressed in units of change in concentration or abundance per unit time]. Each entity in the network is represented as a switch which assumes a particular level of activation depending on the signals it receives. Parameters describing each immune component in the model included an activation threshold above which signals would be perceived, mimicking the actions of high and low affinity receptors. In addition, decisional input weights were used to capture the dynamic response of a given immune component under all possible combinations of input signals. To accommodate often sparse and partially observed experimental data we redefined the conventional goal-directed search for parameter values to one where resting state and time course data were applied as constraints; combinations of parameter values were retained if they supported model predictions that complied with observed experimental data (35, 36). Regulatory parameters for the immune network model were selected based on adherence to a qualitative summary of results previously reported by our group (Supplementary Table 1). All reference data was projected onto a discrete qualitative scale of relative immune activation (e.g., Low, Nominal, and High). The activation level of each immune component at the NOP resting state was presumed to represent the normal homeostatic range of activation during health as captured by samples acquired during asymptomatic measurement time points and was hence used as a baseline. The dynamic range of each immune component was considered independent from that of all others, such that activation levels were expressed in relative terms and can only be directly compared to a baseline value for that entity alone. Therefore, as an example, an increase in the level of TNF from a relative expression of 1 at rest to a value of 2 during AOM (50% of its dynamic range) should not be taken to signify that its change in absolute concentration in pg/ml would necessarily be equal to that of another cytokine given the same relative increase.

The identification of appropriate values for the model parameters dictating the dynamic behavior of the network was performed using a constraint satisfaction approach refined by our group and implemented as a software tool for the analysis of biological networks (BioMC) (34). This search for optimal parameter values enforced strict compliance to stable immune resting states and maximized adherence to transient response data while also minimizing model complexity. The degree of adherence to reference data was expressed as an error score, where the predicted response at each timepoint was subtracted from the input constraint at the corresponding timepoint. Note that greater departure is possible for immune entities with multiple activation levels. Maximum departure from a binary network entity (with possible values of 0 and 1) is 1, while maximum departure from a ternary node (with possible values of 0, 1, and 2) is 2. Overall error for each candidate model was the sum of the deviation over all predicted immune responses expressed as a fraction of the maximum possible error, where 0% error signified perfect adherence and 100% error signified complete failure to adhere.

### Experimental Validation Data

To test the validity of initial candidate immune network models we used cryopreserved serum samples from 142 children (94 NOP, 48 sOP) aged 6–30 months (median 14.3 months, IQR 12.1–15.5) previously collected under a protocol approved by the Institutional Review Boards of Rochester General Hospital and the University of Rochester. Samples within this age range were specifically chosen to study the immune system when sOP children are at their highest risk of experiencing infection (1). Serum levels of IL-4 and CXCL8 were measured on a Bio-Plex 200 machine (Bio-Rad) using a multiplex ELISA kit (EMD Millipore) according to the manufacturer's instructions. Care was taken to account for the frequency with which these children experienced viral upper respiratory infections (URI) which did not progress to AOM.

### Assessment of Immune Network Model Structure

In situations where predictions from even the best candidate immune network model continued to deviate from observed experimental data, the basic structure of the network was assessed for deficiencies. We applied a method proposed by Guimerà and Sales-Pardo wherein the connectivity of the putative network was evaluated for compliance with known properties of immune networks and tested for possible absence of necessary regulatory actions (false negatives) as well as the inclusion of spurious regulatory actions (false positives) (37). We applied this algorithm to estimate the likelihood that additional regulatory actions were required for the entities with the greatest departure from input data. The newly suggested actions, initially absent from the immune network, were included and the logical parameters re-estimated for the revised network.

### Statistics

Statistical analyses of predicted immune network trajectories and cytokine measurements were performed in R version 3.4.2 (38). Steady state predictions were compared across sOP and NOP phenotypic groups using the Wilcoxon rank-sum test. Predicted response trajectories were compared using two-way ANOVA for an otitis proneness group effect and progression across time. Where appropriate, *p*-values were adjusted for multiple comparisons using the Benjamini-Hochberg correction for a 5% false discovery rate. Serum cytokine levels were analyzed using linear models accounting for otitis proneness, frequency of viral URIs not progressing to AOM, and interactions between these terms. Figures were prepared using ggplot2 in R (39).

## Results

### Network Construction

**Figure 2:**
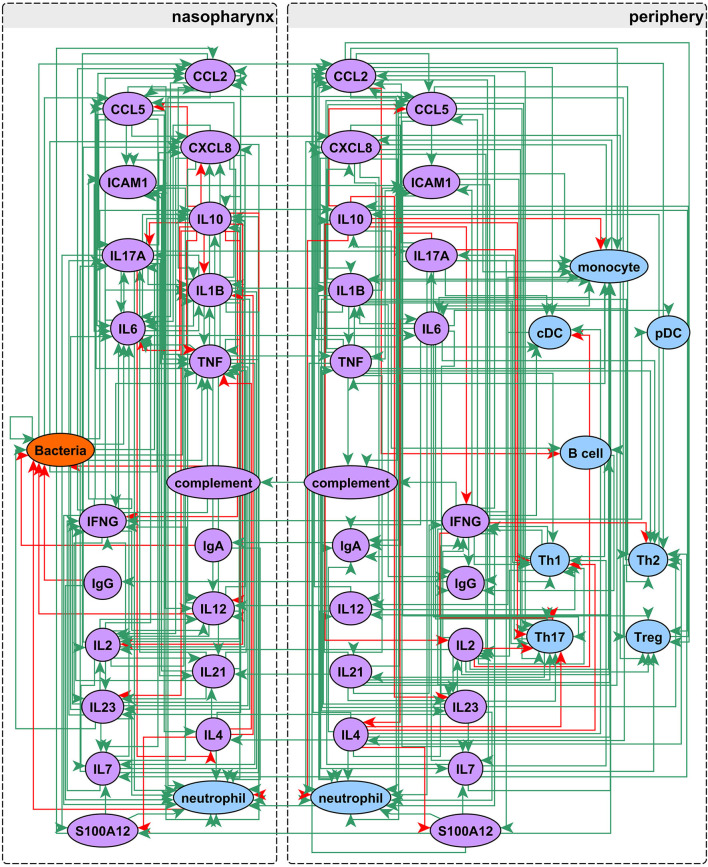
Network assembly. Network structure shows segregation into nasopharyngeal and peripheral compartments with interactions between cell populations, immune mediators, and bacterial infection. The network possesses 51 entities and 320 edges: green edges activate and red inhibit their targets.

Our network construction method is a sub-discipline of systems biology. It uses concepts of electrical circuitry with on and off “switches” (nodes) connected by positive and negative “wires” (interaction edges). Immune cells and functional mediators are represented nodes whose behavior is governed by the known mechanistic interactions between them, resulting in activation or suppression. The network construction is based on documented regulatory interactions from published scientific literature. Based on known information about the network structure (nodes and edges) and its behavior observed in a particular biological context, the regulatory parameters governing the network can be deduced. Using this approach we sought to understand differences in immune response between sOP and NOP children in early childhood. Our goal was to use this approach to identify the most likely central immune mechanisms responsible for otitis proneness vs. otitis resistance. The approach succeeded in this regard, pointing to a specific limited experiment involving measuring two cytokines in a single body compartment that would further reduce the likely responsible immune mechanistic differences between the populations. Thus, we describe a novel, network biology-driven modeling method that is responsive to an iterative process to assist in guiding experiment planning with a goal of identifying the most central immune mechanisms responsible for clinical differences in otitis infection susceptibility. We anticipate this process will identify the best options for therapeutic intervention. As shown in Figure 1 we reviewed published literature concerning pediatric immune responses to bacterial AOM and identified components of the immune system involved in AOM pathogenesis and resolution. Regulatory interactions between these components were identified in the Pathway Studio database, a compendium assembled through automated text mining (30) (see Materials and Methods). To account for the anatomical separation between the NP mucosal and peripheral blood compartments, the overarching network structure was segregated into mucosal “nasopharynx” and systemic “periphery” subnetworks (Figure 2), applying principles developed for analysis of discrete logical networks with spatially-distinct components (40, 41). Interactions between immune cell populations were limited to the peripheral subnetwork (with the exception of neutrophils, which were modeled in both compartments). Cytokines documented as being directly affected by bacterial infection were included in the model of immune response in the NP, and could influence systemic immune signaling. Peripheral cytokines could likewise influence the NP immune response the resulting immune network comprised 51 immune cell and cytokine/protein entities with 320 regulatory connections (edges). The NP compartment contained 22 entities and 138 edges, and the periphery compartment contained 29 entities and 161 edges. The remaining 21 edges were dedicated to communication between these compartments. The network edges were supported by 20,844 published references (median 14 references per edge).

### Defining Model Constraints From Experimentally Observed Behaviors

We collated 12 years of experimental observations from our studies of sOP and NOP children into a set of reference immune response trajectories defining the course of an AOM episode from health to acute disease. Results of experiments where PBMCs from these same children were subjected to experimental stimulation *in vitro* were also included in the set of experimentally observed results. Relative activation of each entity (e.g., cytokines, cell populations, and bacterial infection) was represented qualitatively as a discrete value such that higher values indicated increased activation. Where experimental observations did not support a significant difference in expression across groups or with time for an individual marker taken in isolation, that marker was not used to constrain the immune network-predicted behavior. Instead, activation levels for these “unobserved” entities were predicted as dictated by the logical parameters of the regulatory network model. For example, while circulating ICAM1 was identified as part of a diagnostic biomarker for AOM in our prior studies by providing context to S100A12 and IL10 expression (6), it did not itself vary significantly across groups when considered in isolation. Hence, peripheral ICAM1 expression was not used to constrain network models to consider; rather its activation was predicted in the context of the other immune cells and cytokines/proteins.

Our literature search yielded published references with observations for 46 of the 51 network nodes (immune cells and cytokines/proteins), with no well-defined observations available for ICAM1, IgA, IL7, IL21, and IL23 in the periphery subnetwork. These were nevertheless retained in the network model to enable better representation of peripheral immune function. Of the 51 network nodes, 29 were represented as binary variables and 22 were represented as ternary variables as supported by the experimental data (nodes without experimental data to support them as binary or ternary were represented as binary). The experimental observations for these 51 network nodes are depicted in Supplementary Figure 1 and full description with reference support in Supplementary Table 1.

To better represent the homeostatic regulation of the immune system, we made the following assumptions. Resting states for sOP and NOP (defined by samples collected during regularly-scheduled healthy visits of children where no AOM symptoms were present) were defined as dynamically stable states, such that the system would remain in either of those states unless disturbed; and that following resolution of infection the immune system would return as closely as possible to its prior homeostatic resting state. Since *in vitro* PBMC cultures do not possess similar homeostatic mechanisms, this constraint was not applied to the trajectories derived from PBMC experiments (Supplementary Figure 1B). Exact compliance with the NOP and sOP phenotypic resting states was strictly enforced, while predictions of transient response during AOM pathogenesis, recovery, and *in vitro* stimulation were constrained to match the published reference data along these trajectories as closely as possible. During healthy rest, there were 28 observed immune cell and/or cytokine/protein variables for NOP and 19 for sOP that served as hard constraints to be matched exactly. During AOM, there were 32 observed immune cell and/or cytokine/protein variables for NOP and 24 for sOP. *In vitro* experiments with various TLR ligands and heat-killed bacteria were modeled with B cells, T cells, and antigen-presenting cells activated to their maximum extent. Separate trajectories represented NOP and sOP PBMC responses. Since these experiments were performed with PBMCs in the absence of any interaction with the NP compartment, the immune responses during those experiments were modeled as inactive in the NP compartment. *In vitro* stimulation provided data on 44 immune cell and/or cytokine/protein variables for NOP and 38 immune cell and/or cytokine/protein variables for sOP responses. Following stimulation, there were changes in 40 immune cell and/or cytokine/protein variables for NOP and 34 immune cell and/or cytokine/protein variables for sOP immune responses.

### Enforcing Adherence of Predicted Immune Response to Observed Experiments

**Figure 3:**
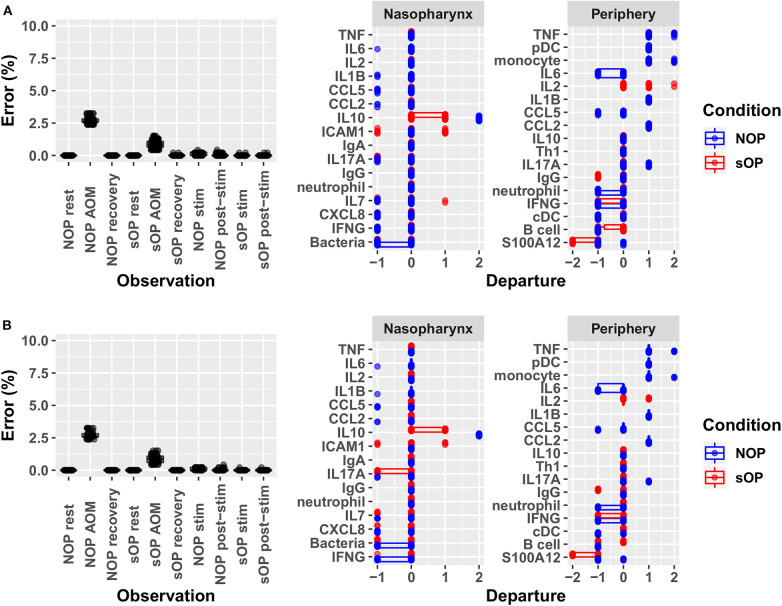
Identifying sources of error. Deviation from observed behavior for **(A)** the 82 candidate models with <5% overall error was decomposed into deviation across markers at each of the 10 experimental conditions used to constrain model identification (left panel) and into deviation across all conditions for constrained markers during acute AOM (right panel). A similar decomposition of error was presented for **(B)** the 44 candidate models remaining after validation against new experimental measurements. Departure indicates the magnitude and direction (positive or negative) of the error contributed by each network entity. Error is computed as the ratio of discrete deviations across all markers to the overall maximum possible deviation.

Adherence to prior experimental reference data in the NP and systemic compartments served to reduce the number of feasible models. Recall that each candidate immune network model consisted of two basic components: a circuit map of the network connectivity and a set of parameter values describing the flow of information through the network, or its kinetics. As such, any given model constitutes a fully deterministic ensemble of transition rules dictating the dynamic response of the immune network under a given set of conditions. Model parameters including activation thresholds and weights dictating the logical rules for the resolution of competing input signals were derived using a constraint-satisfaction formalism as previously described (35). Model parameterization was conducted with the aim of enforcing strict compliance with known stable states while also minimizing divergence from transient states observed experimentally during response to AOM infection *in vivo* and *in vitro* polyclonal stimulation of PBMCs. We favored models where the parameter sets defined an immune network that supported the transition from one experimental observation to the next with the smallest number of intermediate steps. In other words, we searched for the most efficient immune signaling kinetics, which give rise to the shortest possible response trajectories (34, 36). With the available prior experimental reference data (Supplementary Figure 1) and given these model identification criteria we obtained 82 candidate immune network models which adhered to previously observed immune response behavior with <5% overall error across all observations. The main sources of error were the response at onset of AOM for sOP and NOP populations, with the error for individual observations under these conditions ranging from 1 to 3% (Figure 3). Since the predicted responses at onset of AOM constituted the major source of remaining error, we examined these predictions in detail to identify the specific entities most poorly matching the reference data. In particular, activation levels of TNF and monocytes in the NOP peripheral compartment were poorly reproduced by all models, with some departing from the reference data to the full available range. Similarly, predictions of peripheral S100A12 activation at onset of AOM among sOP children deviated from the expected values by as much as the full range of measured values (Figure 3A). These deviations in expected behavior in TNF, monocyte and S100A12 activation suggested that the basic immune network model circuit might be missing additional regulatory actions involving these biological entities, since the predicted activation of all other entities matched the reference data in at least one model.

### Verification of Model Structure

To address the possibility of inadequacies in the immune network model structure, the overall connectivity of the signaling network was evaluated further for potentially missing (false negatives) or spurious (false positive) regulatory actions based on generalized characteristics of network structure (37). Since the two network entities with the greatest departure from reference data were peripheral TNF and monocyte activation, we focused on potential missing regulatory connections to these in particular. This analysis of network structure did not suggest that any additional regulatory connections to peripheral monocytes were required, as the predicted confidence score associated with the inclusion of any such connections was 56% or lower. An additional edge connecting Th17 cells to TNF was suggested with a reliability score of 71%, but in this case the predicted TNF expression was higher than expected, indicating a potential need for a negative regulator. Since Th17 cells are known producers of TNF (42, 43), the biological plausibility of this suggestion in this context was questionable.

In the absence of obvious deficiencies in immune network model structure, the next important source of potential error consisted of inappropriate encoding of the data. It is important to recall that these deviations were greatest in the case of observed episodes of AOM. This should not be entirely unexpected. Samples used in the assembly of experimental reference constraints were collected at varying points in time during the course of an AOM episode (onset was defined by the child being brought to a health care provider and that might well vary by several days depending on the parent of the child), introducing additional variability. Interestingly, despite this inherent variability in the data, model predictions remained consistent with the vast majority of available immune data across all other experimental conditions. The challenge therefore consisted not so much in reducing prediction error further as distinguishing which of the 82 candidate immune network models were the most biologically plausible.

### A First Validation and Model Subset Reduction

**Figure 4:**
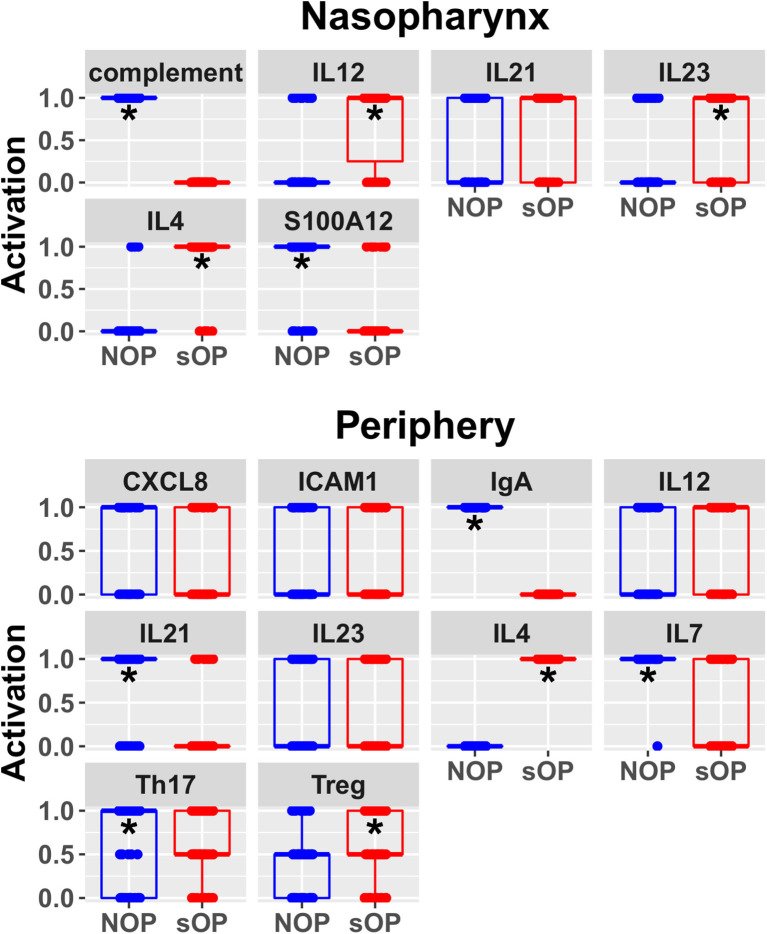
Predicted rest states from all candidate models. Predicted activation at rest for network entities unconstrained during AOM in the nasopharynx and periphery compartments. Each point represents the predicted activation from one candidate parameter set; points were jittered to reduce overlap (*p < 0.05 by BH-corrected Wilcoxon test; stars denote the group with the higher median).

In order to better understand the mechanistic underpinnings of sOP, it was important to reduce the number of competing immune network models that adhered to the existing data. This could only be achieved by adding new data through the assessment of unobserved immune measurements, whether from existing cryopreserved samples or newly acquired samples. Since a sizable biorepository existed, we chose to measure unobserved immune markers in existing samples. In a partial validation of these models, we focused on two basic situations: one where consensus existed across all models regarding a marker's predicted expression levels and one where predictions clearly delineated between large subsets of competing models (Figure 4). IL4 was selected as a marker where all 82 candidate models unanimously predicted elevated expression in the sOP phenotype. We reasoned that agreement with this prediction would serve as a first partial validation of the general viability of the overall immune network model set. CXCL8 was selected as an unmeasured marker where subsets of models disagreed and where the outcome of experimental verification could serve to refine the available solutions by eliminating a significant fraction of the 82 competing models.

**Figure 5:**
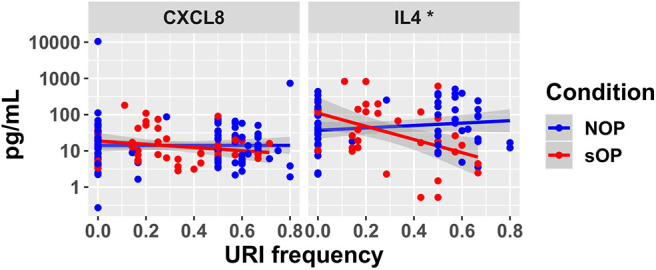
New serum cytokine measurements. Serum levels of cytokines predicted to differ by the top models, plotted against the frequency with which subjects experienced viral URI that did not progress to otitis media. The sOP condition was associated with a significant positive effect on serum IL4 at low viral URI frequencies, while the interaction with viral URI frequency was associated with a significant decrease (*p < 0.05). Neither sOP condition nor viral URI frequency was significantly associated with changes in CXCL8.

In sOP subjects, a significant dependency of IL-4 expression on the frequency with which these children experienced viral URI was observed, such that sOP samples were likely to have higher IL4 levels at low frequencies of viral URI infection (Figure 5, discussed further below). No significant effects of clinical phenotype or URI frequency on circulating CXCL8 levels were identified. Predictions for serum IL4 measurement were consistent with newly acquired data. Measurements of CXCL8 in sOP and NOP subjects (either unanimously active or unanimously inactive) did not find significant differences; this result was consistent with only 44 of the 82 models. Thus, new experimental measurement of only 2 previously unobserved markers reduced the pool of candidate models by approximately half, to 44 competing immune network models.

### Incremental Refinement in Model Predictions

**Figure 6:**
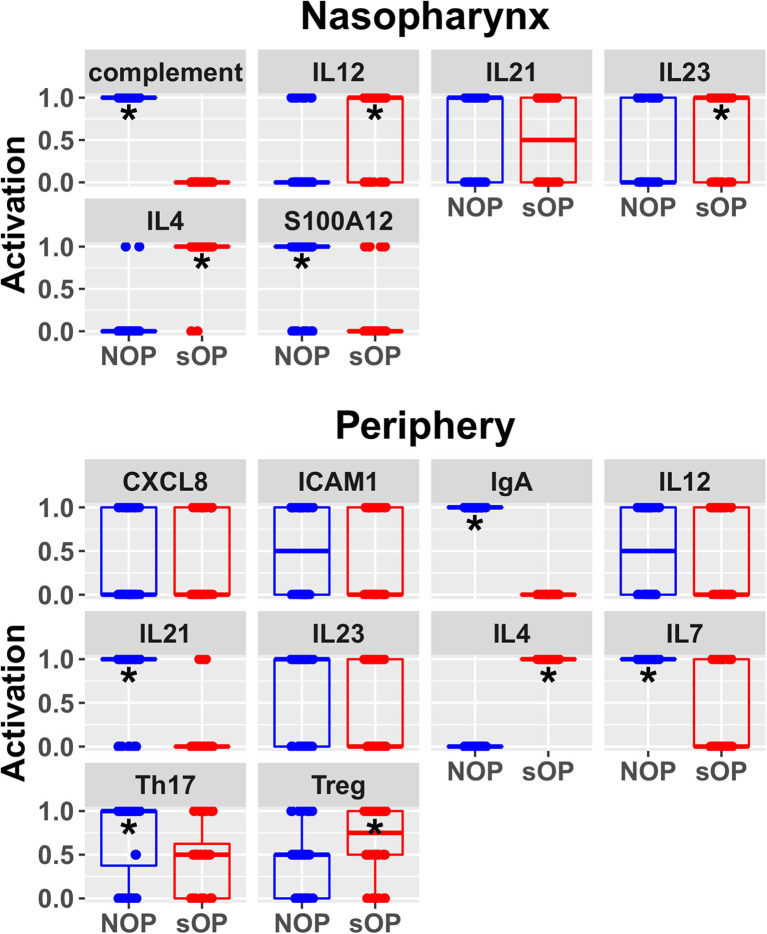
Refined steady state predictions. Activation of unconstrained entities in the nasopharynx and periphery predicted by the subset of 44 validated models (*p < 0.05, BH-corrected Wilcoxon test; stars denote the group with the higher median).

**Figure 7:**
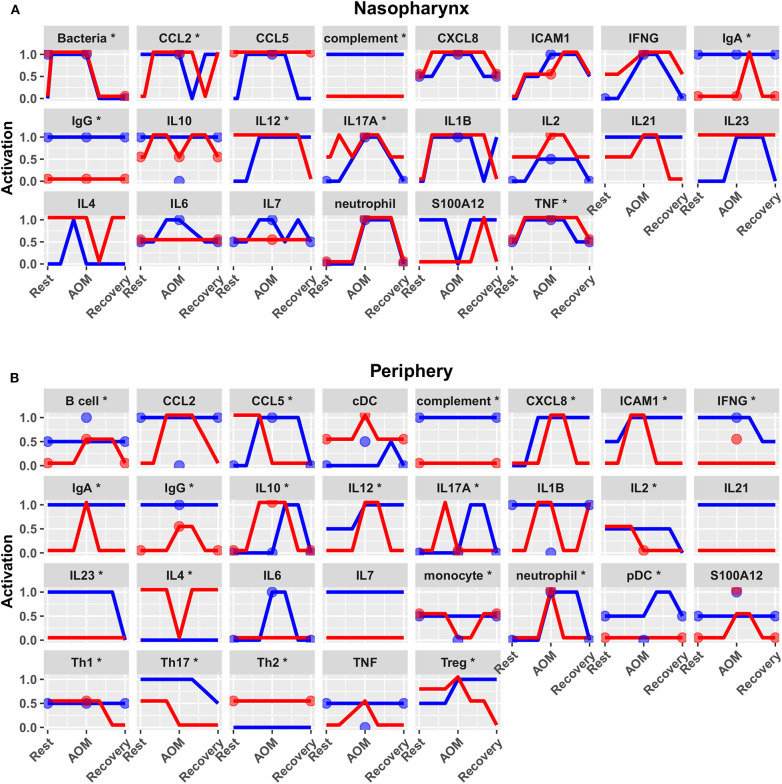
Predicted response trajectories during an episode of AOM. Output trajectories aim to fit the available data on activation levels at rest, AOM, and recovery states as well as predicting the course of events during the processes of pathogenesis and recovery. Responses adhered closely to available reference constraints and additionally predicted behavior of unmeasured entities. Lines show medians of the 44 refined models for each entity in **(A)** nasopharynx and **(B)** periphery compartments, interpolating the predicted response dynamics during the course of AOM pathogenesis and recovery (*p < 0.05 for interaction between otitis-prone condition and infection stage by BH-corrected ANOVA during dynamic response). Dots show reference data where available. Blue, NOP; red, sOP.

Predicted activation of unconstrained immune network nodes during healthy rest in the reduced subset of 44 competing immune network models is shown in Figure 6. These are consistent with predictions from the full pool of 82 candidate models (Figure 4). The models in the reduced pool fit new experimental data *in addition* to the original reference constraints (Supplementary Figure 1), yielding a more rigorously constrained set of remaining candidate models. Predictions over the entire period of the simulated trajectory show a high degree of consensus that AOM pathogenesis and resolution proceeds differently over time in the sOP compared to the NOP phenotype (Figure 7). In particular, the predicted response for almost every cell type represented in the peripheral subnetwork of the model (B cells, monocytes, neutrophils, pDC, and T cells, with the sole exception of cDC) showed significant variability with respect to the interaction between the otitis-prone condition and the stage of the response. This includes all four of the modeled T cell subpopulations. In general, the peripheral compartment displayed impaired adaptive immunity in sOP children that is sustained through AOM pathogenesis, suggesting mobilization of memory to AOM-causing bacteria may be deficient in these children. In contrast, Th2 cells were higher and sustained in the periphery of sOP children during AOM, suggesting immune deviation to a non-protective Th2 response may be a factor in the susceptibility of sOP children to repeat viral-bacterial co-infections. Deficient adaptive immunity in the periphery is likely influencing the NP response to infection, as several inflammatory cytokines and chemokines were higher at onset and/or sustained longer in sOP children during AOM pathogenesis, including CCL2, IL-12, IL-17A, and TNF-a.

### Phenotype-Specific Early Activation of Immune Response Subsets in sOP

**Figure 8:**
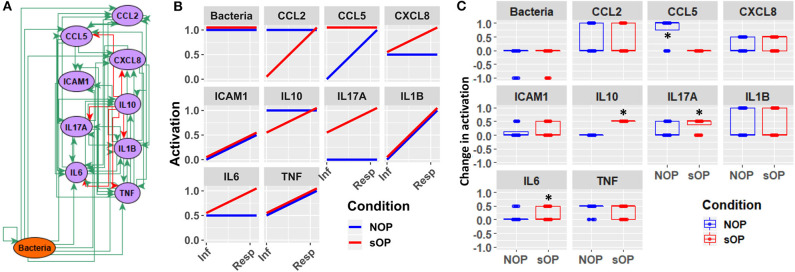
Early response motifs. **(A)** Network entities initially engaged by bacterial infection during AOM pathogenesis in the nasopharynx. **(B)** Absolute activation of these early response elements immediately following bacterial infection: lines show medians for the 44 refined models. **(C)** Relative change in activation compared to resting levels early after bacterial infection in NOP and sOP settings, normalized to maximum activation for each entity (*p < 0.05, BH-corrected Wilcoxon test; stars denote the group with the higher median).

In addition to the predicted immune status for resting states in sOP and NOP, we further analyzed the expected responses triggered by bacterial infection during the early stages of AOM pathogenesis. We expect these events to be limited to the nasopharynx (Figure 8A). By comparing the change in activation predicted by each of the remaining 44 candidate immune network models, we could identify mechanistic differences in the response to bacterial infection. We found significant differences in predicted changes in the activation of NP CCL5, IL10, IL17A, and IL6 (Figure 8B). Specifically, most models predicted increased CCL5 early in the NOP but not sOP response, bringing NOP CCL5 to the constitutive high level predicted for sOP CCL5. Conversely, IL10, IL17A, and IL6 were predicted to increase early only in the sOP response. In the case of IL10, the NOP phenotype was predicted to show constitutive high activation which was only transiently equaled by the sOP response to infection. IL17A was predicted to be generally elevated in the sOP NP, undergoing an especially early activation upon bacterial infection. Finally, NP IL6 was eventually activated in both sOP and NOP phenotypes, but this occurred earlier in sOP. The general sOP profile in this NP immune network model could be characterized as pro-inflammatory, in that inflammatory markers were predicted to be overexpressed early in infection while IL10 was consistently reduced. Importantly, these model outcomes point to specific future experiments to identify key immune mediators that have differentiate NP immune function in sOP and NOP children.

## Discussion

While mathematical models have been used to study immune response, their use in otitis media has been limited to engineering models of biofilm formation (44) and the use of conventional pharmaco-dynamic modeling of response to antibiotic agents (45) or vaccine efficacy (46). Here we construct a mechanistically-informed model of immune response from prior knowledge and test alignment of these known immune dynamic responses with experimental observation. Drawing on a broad base of published knowledge, we constructed a novel immune network model involving immune cells and cytokines/proteins that mediate immune signaling involved in the pathogenesis of AOM in young children who are especially prone to such infections. We show the value of model predictions to guide targeted experimentation to refine understanding of differing immune mechanisms engaged in disease pathogenesis in more susceptible individuals and to generate additional high-level hypotheses for future study. Specifically, we derived a reduced set of immune network models that predict an immune profile in the peripheral compartment of sOP children consistent with a Treg-dominated phenotype, as well as indications of a chronic inflammatory profile in the sOP nasopharynx.

A basic and central finding of this work is that both sOP and NOP immune profiles can be accommodated by a single common assembly of immune cells and protein mediators, some of which diverge in mechanistic activation. This suggests that susceptibility to AOM may in part result from a combination of environmental factors, such as infection history and/or interactions within the nasopharyngeal microbiota (47), that induce a self-sustaining immune dysfunction in an initially healthy respiratory immune system. In other words, there is no essential “damage” to the regulatory circuitry governing immune responsiveness, as the sOP phenotype can be represented as an alternative immune regulatory program available to the same mechanisms that support the NOP phenotype, without altering the underlying immune circuitry. The ready availability of candidate models that adhere to available experimental reference data and support both sOP and NOP as stable persistent phenotypes lends credence to this notion, and raises the possibility that the sOP phenotype may be amenable to immunomodulatory therapeutic intervention.

The iterative process we used to construct and revise plausible immune networks active in sOP and NOP child responses to AOM infection (Figure 1) has broad potential applications. Based on a common network of immune signaling mechanisms, we initially found 82 candidate immune network parameter sets, each of which adhered equally well to available published data in a subset of immune markers measured during health and AOM in sOP and NOP child populations as well as *in vitro* experiments. Predictions from these models for immune cell and molecular readouts identified specific experiments that had the potential to invalidate competing models and refine the model pool. When we measured circulating levels of two molecules (IL4 and CXCL8), where model predictions were in broad agreement or disagreement, respectively, the pool of models was reduced by approximately half. This simple experiment demonstrates the significant potential of using coarse-grained computational models to design focused and insightful experiments in a way that rapidly reduces a pool of competing mechanistic hypotheses to the most biologically plausible candidates. In addition, the experiment helped identify an unexpected association of serum IL4 levels with viral URI history independent of bacterial AOM, in that increased viral URI frequency was negatively associated with IL4 level in sOP children. This was an intriguing result, since bacterial AOM can be understood as a complication of concurrent viral URI (48). With regard to the pathology itself, these results highlight the importance of viral infection history, and point to the possibility of broader immunological consequences of sOP status in addition to frequent ear infections. Moreover, in light of the influence of the precipitating viral infection on the course of an AOM episode (9, 10), the differences in early activation of immune responses predicted by our candidate models (Figure 8) may be capturing residual effects of viral URI. Accordingly, in future work, we will direct our efforts toward modeling viral URI explicitly.

The approach to model construction presented here utilizes a large number of published observations (using Elsevier Pathway Studio) in concert with primary data observations from our laboratory. Therefore, model predictions are robust and based on a large number of interacting factors in each case. For instance, the new prediction that Th2 responses are higher and sustained in sOP children during AOM is derived from analyses of multiple input (CCL2, CCL5, IFNG, IL1B, IL23, IL4, IL6, IL7, and TNF) and output (IL4, IL6, and IL10) signals over the course of an AOM event. Therefore, this robust prediction provides a strong premise for follow-up studies on the impact of Th2 immune responses in children prone to repeat AOM. Using an overlapping but distinct algorithm, Treg responses were also predicted to be higher in the periphery of sOP children. Although accumulation of Tregs is often associated with chronic inflammation in extralymphoid tissues, our prior data also suggested increased *foxp3* mRNA in sOP children. AOM is an acute infection event that typically resolves in a few days. However, it is possible that in sOP children, repeat AOM infections by related pathogens may induce similar changes in immunity to those in chronic inflammatory conditions, i.e., chronic innate inflammation and increased Tregs. More work is needed to better understand how these conditions relate in children.

New insights and hypotheses emerge from this work. The modeling and validation studies suggest a number of possible immune-related mechanisms supporting recurrent AOM susceptibility. Given the high complexity of the expression patterns and dynamic regulation of cytokines and their receptors, we group these potential mechanisms together for the sake of discussion here concerning future directions. First, the predicted differences in IL7 levels between sOP and NOP (Figure 6) may point to differential maintenance of B and T cell memory in these populations. Our prior published data showed diminished circulating T and B cell memory in sOP compared to NOP children (16, 22, 25, 49). IL7 modulation is one mechanism that could be explored to further understanding of our prior observations regarding poor maintenance of B and T cell memory in sOP children. Second, analysis of the data strongly suggests that cytokine-driven differences in T and B cell differentiation play a pivotal role in AOM susceptibility. IL4 is a key driver of Th2 immune responses, and regulates B cell survival, Ig secretion and MHC Class II expression (50, 51). This may account for the observed Th2-dominant phenotype in sOP children. IL12 orchestrates Th1 immune responses and can also act as a regulator of T follicular helper (Tfh) development (52, 53). Since it was observed to be increased in sOP children in the NP only, it is possible that NP macrophages and dendritic cells are producing high levels of IL-12 compared to NOP children. IL21 is secreted by Th17 and Tfh cells and also functions in their development (54, 55). Low peripheral IL21 may be connected to the lower antibody levels observed in sOP child serum. Regulation of Th17 and Treg populations was identified in prior experiments as a potential mechanism at play in distinguishing sOP and NOP immune responses (22). Results in the modeling were consistent with altered Th17 immune responses. Th17 responses are critical for protection from both Spn and non-typeable *Haemophilus influenza* infections, the two major causative agents of AOM (18, 56, 57). Reciprocal regulation of Th17 and Treg cells is well-documented (58–60). Pneumococci impact host immunity by promoting Treg dominance to support prolonged NP carriage (20, 61), which further suggests TGFβ as a candidate for inclusion in future iterations of the model because TGFβ could be implicated in altered Th17/Treg balance. Interestingly, dysregulation of the TGFβ pathway at the genomic level has been implicated in susceptibility to otitis media in children in both Australia (62) and Greece (63).

In conclusion, we have demonstrated that a clinical phenotype among young children—otitis proneness—can be explained in terms of well-documented mechanisms as a self-sustaining regulatory regime characterized by reduced homeostatic production of pro-inflammatory and anti-inflammatory cytokines, consistent with a pattern of Th2 and Treg skewing. Our immune network models and simulations suggest that sOP and NOP children experience AOM differently because they are executing alternative immune regulatory programs governing both stable homeostasis and immune response, while the fundamental mechanisms and network remain unaltered. This further raises the possibility that sOP children may be treated by immunomodulatory intervention to reverse or rescue from the sOP state.

## Data Availability Statement

All datasets generated for this study are included in the article/Supplementary Material.

## Ethics Statement

The studies involving human participants were reviewed and approved by Institutional Review Boards of Rochester General Hospital and the University of Rochester. Written informed consent to participate in this study was provided by the participants' legal guardian/next of kin.

## Author Contributions

MM and TC assembled the data, designed the biological model, contributed biological interpretations of the results, and drafted the initial manuscript and its subsequent revisions. MM ran simulations, performed experiments, and prepared the graphical results. GB and MP directed the work, reviewed the results, and contributed directly to the writing and editing of the manuscript. All authors read and approved the final manuscript.

## Conflict of Interest

The authors declare that the research was conducted in the absence of any commercial or financial relationships that could be construed as a potential conflict of interest.

## Supplementary Material

Supplementary File Contains (i) Supplementary Table 1 describing reference constraints derived from the data and used for model parameter identification, and (ii) Supplementary Figure 1 describing the qualitative expression levels of immune markers observed across trajectories used to constrain the model.

## Abbreviations

AOM: acute otitis media
cDC: classical dendritic cells
CXCL: C-X-C Motif Chemokine Ligand
IL: interleukin
Mcat: *Moraxella catarrhalis*
NLP: natural language processing
NOP: non-otitis prone
NP: nasopharyngeal
NTHi: non-typeable *Haemophilus influenzae*
Ig: immunoglobulin
OP: otitis prone
PBMC: peripheral blood mononuclear cells
pDC: plasmacytoid dendritic cells
sOP: stringently defined otitis prone
Spn: *Streptococcus pneumoniae*
STG: state transition graph
TGF: transforming growth factor
Th: T helper cell
TNF: tumor necrosis factor
Treg: T regulatory cells
URI: upper respiratory infection.
