# Supplementary material for: Immune Network Modeling Predicts Specific Nasopharyngeal and Peripheral Immune Dysregulation in Otitis-Prone Children
Source: Front Immunol. 2020 Jun 11;11:1168. doi: 10.3389/fimmu.2020.01168 (PMC7301607; doi:10.3389/fimmu.2020.01168)
Supplement: Supplementary File — Contains (i) Supplementary Table 1 describing reference constraints derived from the data and used for model parameter identification, and (ii) Supplementary Figure 1 describing the qualitative expression levels of immune markers observed across trajectories used to constrain the model. [file Data_Sheet_1.docx]

|  | System |  | in vivo (Child samples) | | | | in vitro (Child PBMC) | | | | | |
| --- | --- | --- | --- | --- | --- | --- | --- | --- | --- | --- | --- | --- |
|  | Condition |  | NOP | | sOP | | NOP | | | sOP | | |
| Module | Entity | Max | rest | AOM | rest | AOM | unstim | stim | post-stim | unstim | stim | post-stim |
| **NP** | Bacteria | 1 | 0 | 1 | 0 | 1 | 0 | 0 | 0 | 0 | 0 | 0 |
|  | CCL2 | 1 |  | 1(12, 60) |  | 1(12, 60) | 0 | 0 | 0 | 0 | 0 | 0 |
|  | CCL5 | 1 |  | 1(60) | 1(9, 60) | 1(60) | 0 | 0 | 0 | 0 | 0 | 0 |
|  | complement | 1 |  |  |  |  | 0 | 0 | 0 | 0 | 0 | 0 |
|  | CXCL8 | 2 | 1(9) | 2(9, 12) | 1(9) | 2(9) | 0 | 0 | 0 | 0 | 0 | 0 |
|  | ICAM1 | 2 |  | 2(12) |  | 1(12) | 0 | 0 | 0 | 0 | 0 | 0 |
|  | IFNG | 2 | 0(61) | 2(61) |  | 2(61) | 0 | 0 | 0 | 0 | 0 | 0 |
|  | IgA | 1 | 1(62) | 1(62) | 0(62) | 0(62) | 0 | 0 | 0 | 0 | 0 | 0 |
|  | IgG | 1 | 1(62) | 1(62) | 0(62) | 0(62) | 0 | 0 | 0 | 0 | 0 | 0 |
|  | IL10 | 2 | 2(60) | 0(60) | 1(60) | 1(60) | 0 | 0 | 0 | 0 | 0 | 0 |
|  | IL12 | 1 |  |  |  |  | 0 | 0 | 0 | 0 | 0 | 0 |
|  | IL17A | 2 | 0(61) | 2(60, 61) |  | 2(60, 61) | 0 | 0 | 0 | 0 | 0 | 0 |
|  | IL1B | 1 |  | 1(12, 60) |  |  | 0 | 0 | 0 | 0 | 0 | 0 |
|  | IL2 | 2 | 0(61) | 1(60, 61) |  | 2(60, 61) | 0 | 0 | 0 | 0 | 0 | 0 |
|  | IL21 | 1 |  |  |  |  | 0 | 0 | 0 | 0 | 0 | 0 |
|  | IL23 | 1 |  |  |  |  | 0 | 0 | 0 | 0 | 0 | 0 |
|  | IL4 | 1 |  |  |  |  | 0 | 0 | 0 | 0 | 0 | 0 |
|  | IL6 | 2 | 1(9) | 2(9, 12, 60) | 1(9) |  | 0 | 0 | 0 | 0 | 0 | 0 |
|  | IL7 | 2 | 1(61) | 2(61) |  | 1(61) | 0 | 0 | 0 | 0 | 0 | 0 |
|  | neutrophil | 1 | 0(9) | 1(9, 12, 61) | 0(9) | 1(9, 12, 61) | 0 | 0 | 0 | 0 | 0 | 0 |
|  | S100A12 | 1 |  |  |  |  | 0 | 0 | 0 | 0 | 0 | 0 |
|  | TNF | 2 | 1(9) | 2(9) | 1(9) | 2(9) | 0 | 0 | 0 | 0 | 0 | 0 |
| **Periphery** | B cell | 2 | 1(16, 63) | 2(14, 15) | 0(63) | 1(14, 15) | 1(16, 63) | 2(64) | 2(64) | 0(16, 63) | 2(64) | 1(64) |
|  | CCL2 | 1 | 1(25, 65) | 0(25, 65) |  |  | 1(66) | 1(66) | 1(66) |  |  | 1(66) |
|  | CCL5 | 1 | 0(65) | 1(65) |  |  | 0(65) | 0(65) |  |  |  |  |
|  | cDC | 2 | 0(24) | 1(25, 65) | 1(24) | 2(24) | 0(22, 66) | 2(66) | 2(66) | 1(22, 66) | 2(66) |  |
|  | complement | 1 | 1(65) |  | 0(65) |  | 1(65) | 1(65) |  | 0(65) | 0(65) |  |
|  | CXCL8 | 1 |  |  |  |  |  |  | 1(66) |  |  | 1(66) |
|  | ICAM1 | 1 |  |  |  |  |  |  |  |  |  |  |
|  | IFNG | 2 |  | 2(25, 61) |  | 1(25, 61) | 0 | 0(61, 66) | 2(22, 64) | 1(61, 66) | 1(61, 66) | 1(22, 64) |
|  | IgA | 1 |  |  |  |  |  |  |  |  |  |  |
|  | IgG | 2 |  | 2(14, 15) | 0(16, 67) | 1(14, 15) | 1(16, 67) | 1(16, 67) |  | 0(16, 67) | 0(16, 67) |  |
|  | IL10 | 1 | 0(26, 65) | 0(26, 65) | 0(26) | 1(26) | 0(26, 65) | 0(26) | 1(22, 66) | 0(22, 66) | 0(26) | 1(22, 66) |
|  | IL12 | 1 |  |  |  |  | 1(66) | 1(66) | 1(66) | 0(66) | 0(66) | 1(66) |
|  | IL17A | 1 | 0(61) | 0(61) |  | 0(61) | 0(22, 61, 64) | 0(22, 61) |  |  |  |  |
|  | IL1B | 1 | 1(25, 65) | 0(25, 65) |  |  | 1(66) | 1(66) | 1(66) |  |  | 1(66) |
|  | IL2 | 2 |  |  |  | 0(61) | 2(61) | 2(22, 64) | 2(22, 64) | 1(61) | 1(22, 64) | 1(22, 64) |
|  | IL21 | 1 |  |  |  |  |  |  |  |  |  |  |
|  | IL23 | 1 |  |  |  |  |  |  |  |  |  |  |
|  | IL4 | 1 |  |  |  |  |  |  | 1(22) |  |  | 1(22) |
|  | IL6 | 1 | 0(25) | 1(25) |  |  | 0(25) | 0(22, 66) | 1(22, 66) |  |  | 1(22, 66) |
|  | IL7 | 1 |  |  |  |  |  |  |  |  |  |  |
|  | monocyte | 2 | 1(24, 25, 65) | 0(25, 65) | 1(24) |  | 1(66) | 2(66) | 2(66) | 1(66) | 2(66) |  |
|  | neutrophil | 1 | 0(65, 68) | 1(65, 68) |  | 1(68) | 0 | 0 | 0 | 0 | 0 | 0 |
|  | pDC | 2 | 1(25, 65, 68) | 0(25, 65) | 0(24) |  | 1(66) | 2(66) | 2(66) | 0(66) | 2(66) |  |
|  | S100A12 | 2 | 1(7, 65, 68) | 2(7, 65, 68) | 0(7, 65, 68) | 2(7, 65, 68) | 0 | 0 |  |  |  |  |
|  | Th1 | 2 | 1(25, 65) | 1(25, 65) |  | 1(64) | 1(25, 65) | 2(22, 64) | 2(22, 64) | 1(22) | 2(22) | 1(22) |
|  | Th17 | 2 |  |  |  |  | 1(22) | 2(22) | 2(22) | 1(22) | 2(22) |  |
|  | Th2 | 2 |  |  | 1(25) |  | 1(25) | 2(25) | 2(25) | 1(25) | 2(25) |  |
|  | TNF | 2 | 1(25) | 0(25) |  |  | 1(66) | 1(66) | 2(22) | 1(66) | 1(66) |  |
|  | Treg | 2 |  |  |  |  | 1(22) | 2(22) |  | 2(22) | 2(22) |  |

**Supplementary Table 1. Reference constraints used for model parameterization.** “Module” indicates the nasopharyngeal and peripheral subnetworks. “Max” is the maximum activation level available to each entity (including 0 such that a maximum of 1 represents a binary entity and a maximum of 2 represents a ternary entity). Numbers in superscript are references for each constraint. NOP and sOP rest states were constrained to be stable. Blank cells were left unconstrained during parameterization.

**
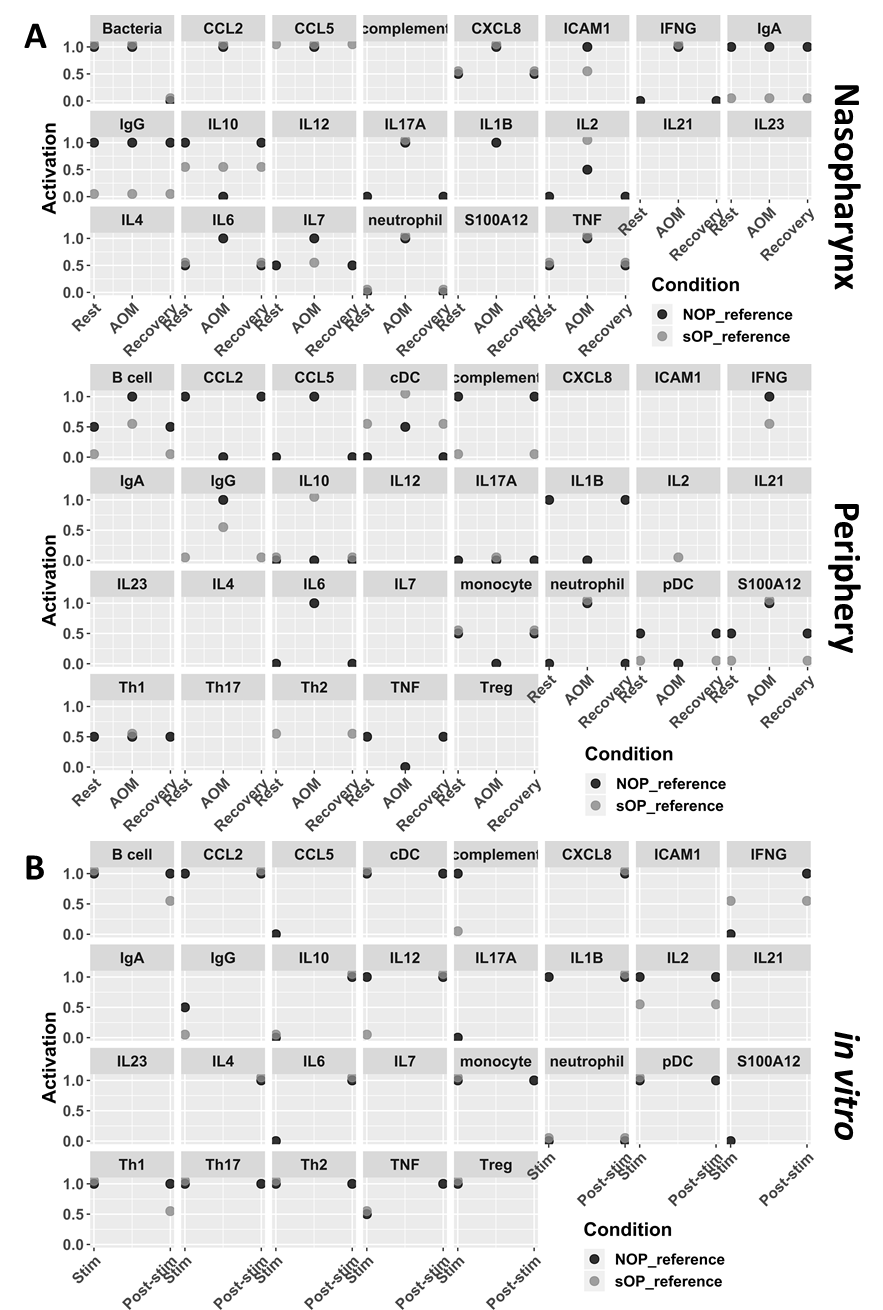
**

**Supplementary Figure 1.** **Qualitative observed trajectories used to constrain the model.** Based on published data, the activation of each entity in the model was expressed as a fraction of its maximum level. **A)** In the nasopharynx and periphery, the activation of each entity was determined during health (rest), AOM episode and post-AOM recovery in order to observed dynamic changes between health and infection. Where possible, entities were assumed to return to their resting levels following AOM. **B)** PBMC responses to polyclonal stimulation in vitro.
